# Investigation of the Electrical Characteristics of Bilayer ZnO/In_2_O_3_ Thin-Film Transistors Fabricated by Solution Processing

**DOI:** 10.3390/ma11112103

**Published:** 2018-10-26

**Authors:** Hyeonju Lee, Xue Zhang, Jung Won Kim, Eui-Jik Kim, Jaehoon Park

**Affiliations:** 1 Department of Electronic Engineering, Hallym University, Chuncheon 24252, Korea; zoozs123@naver.com (H.L.); zhangxue00@naver.com (X.Z.); 2 Department of Environmental Sciences & Biotechnology, Hallym University, Chuncheon 24252, Korea; jwkim@hallym.ac.kr; 3 School of Software, Hallym University, Chuncheon 24252, Korea; ejkim32@hallym.ac.kr

**Keywords:** thin-film transistor, metal-oxide semiconductor, solution process, bilayer

## Abstract

Metal-oxide thin-film transistors (TFTs) have been developed as promising candidates for use in various electronic and optoelectronic applications. In this study, we fabricated bilayer zinc oxide (ZnO)/indium oxide (In_2_O_3_) TFTs by using the sol-gel solution process, and investigated the structural and chemical properties of the bilayer ZnO/In_2_O_3_ semiconductor and the electrical properties of these transistors. The thermogravimetric analysis results showed that ZnO and In_2_O_3_ films can be produced by the thermal annealing process at 350 °C. The grazing incidence X-ray diffraction patterns and X-ray photoemission spectroscopy results revealed that the intensity and position of characteristic peaks related to In_2_O_3_ in the bilayer structure were not affected by the underlying ZnO film. On the other hand, the electrical properties, such as drain current, threshold voltage, and field-effect mobility of the bilayer ZnO/In_2_O_3_ TFTs obviously improved, compared with those of the single-layer In_2_O_3_ TFTs. Considering the energy bands of ZnO and In_2_O_3_, the enhancement in the TFT performance is explained through the electron transport between ZnO and In_2_O_3_ and the formation of an internal electric field in the bilayer structure. In the negative gate-bias stress experiments, it was found that the internal electric field contributes to the electrical stability of the bilayer ZnO/In_2_O_3_ TFT by reducing the negative gate-bias-induced field and suppressing the trapping of holes in the TFT channel. Consequently, we suggest that the bilayer structure of solution-processed metal-oxide semiconductors is a viable means of enhancing the TFT performance.

## 1. Introduction

Metal-oxide semiconductors have been widely studied, given their excellent electrical conductivity and optical transmittance. Thin-film transistors (TFTs) with a metal-oxide semiconductor as the conductive channel layer have also been widely used in various electronic fields. Among them, TFTs based on zinc oxide (ZnO) and indium oxide (In_2_O_3_) have made great progress [1,2,3]. This research has led to many methods being proposed to improve the electrical characteristics and stability of metal-oxide semiconductor-based TFTs. These include ternary and multi-element oxides, such as gallium tin oxide, indium zinc oxide, indium gallium oxide, and indium gallium zinc oxide, which have been used as functional semiconductor layers [4,5,6,7,8,9]. The addition of dopants can also improve the TFT performance, such as In-doped ZnO, Al-doped ZnO, Gd-doped In_2_O_3_, and Ga-doped In_2_O_3_ [10,11,12,13]. A new method using an active bilayer structure has been proposed, and it has been confirmed that the electrical characteristics and stability of the TFT can be improved by using this method [14]. In particular, the bilayer structured TFT uses two metal oxides as the channel layer and combines the advantages of the two semiconductor layers to improve the TFT characteristics. For example, bilayer metal oxide TFTs based on ZnO/ZnO:H, InGaZnO/InGaZnO:Ti, and In_2_O_3_/InGaO have recently been reported [15,16,17].

In this study, we fabricated bilayer ZnO/In_2_O_3_ TFTs by using the sol-gel solution process and investigated the electrical characteristics of these TFTs, compared to those of a single-layer In_2_O_3_ TFT. The prepared films were analyzed by X-ray photoemission spectroscopy (XPS), grazing incidence X-ray diffraction patterns (GIXRD), and field-emission scanning electron microscopy (FE-SEM). The electrical properties of the single- and bilayer TFTs were studied by analyzing the output, transfer, and hysteresis characteristics. The operational stability of the fabricated TFTs was also examined via negative gate-bias stress experiments. The comparative analyses of experimental results enable us to explain how the bilayer ZnO/In_2_O_3_ structure affects the TFT performance.

## 2. Materials and Methods

**Figure 1:**
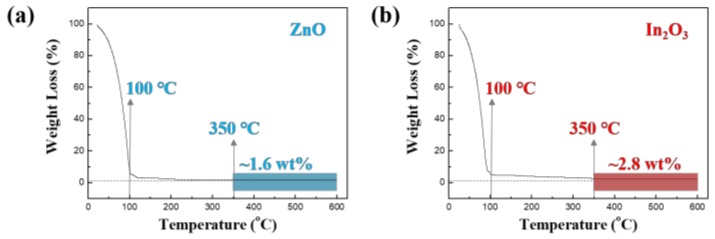
Thermogravimetric analysis (TGA) curves of (**a**) ZnO and (**b**) In_2_O_3_ precursor solutions.

Indium nitrate hydrate (In(NO_3_)_3_∙*x*H_2_O) (300.83 g/mol, Sigma-Aldrich, St. Louis, MO, USA) and zinc nitrate hydrate (Zn(NO_3_)_2_∙*x*H_2_O) (189.40 g/mol, Sigma-Aldrich, St. Louis, MO, USA) were used as precursors in this experiment. The precursor solutions were prepared by dissolving In(NO_3_)_3_∙*x*H_2_O (0.2 M) and Zn(NO_3_)_2_∙*x*H_2_O (0.1 M) in 2-methoxyethanol (CH_3_OCH_2_CHOH) (Sigma-Aldrich, St. Louis, MO, USA). To obtain a homogeneously mixed precursor solution, the prepared solutions were stirred on a hot plate at 75 °C for 6 h by using a magnetic bar. Thermogravimetric analysis (TGA) (N-1000, Sinco, Seoul, Korea) was used to analyze and compare the thermal decomposition processes of the ZnO and In_2_O_3_ precursor solutions. The measurement was carried out by raising the temperature from 25 to 600 °C at a heating rate of 10 °C /min in a nitrogen atmosphere. The TGA curves for the precursor solutions prepared in the experiment are shown in Figure 1. We can see that the initial large weight loss (>90%) of the ZnO and In_2_O_3_ solutions occurs below 100 °C, this being a result of the evaporation of the solvent and the decomposition of the precursor. In this process, the Zn(NO_3_)_2_∙*x*H_2_O and In(NO_3_)_3_∙*x*H_2_O solutions are hydrolyzed to Zn(OH)_2_ and In(OH)_3_, respectively. Thereafter, as the temperature continues to rise, the weight loss of both samples gradually slows and becomes constant. This phenomenon is due to the dehydroxylation process of Zn(OH)_2_ and In(OH)_3_ to form ZnO and In_2_O_3_ [18]; the thermal decomposition of Zn(OH)_2_ to produce ZnO and H_2_O takes place at a temperature of 100–250 °C and that of In(OH)_3_ to produce In_2_O_3_ and H_2_O occurs at 340–850 °C. Furthermore, the weight loss for both precursors is observed to be negligible at temperatures above 350 °C. This indicates that an annealing temperature of 350 °C can be used to convert the precursor solutions into ZnO and In_2_O_3_ films.

**Figure 2:**
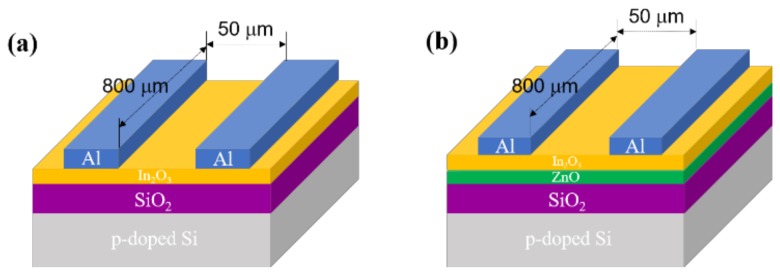
(**a**) Single-layer In_2_O_3_ thin-film transistor (TFT) and (**b**) bilayer ZnO/In_2_O_3_ TFT.

In this study, solution-processed metal oxide TFTs were fabricated on boron-doped silicon wafers with a 100-nm thermally grown silicon dioxide (SiO_2_) dielectric layer. To improve the chemical compatibility between the interface of the SiO_2_ and semiconductor layer, the surface of the cleaned substrate was hydrophilized for 10 s with O_2_ plasma; a radio frequency power of 40 W was applied with an oxygen flow rate of 9 sccm. For the fabrication of the single-layer In_2_O_3_ films, the 0.2 M In_2_O_3_ precursor solution was filtered using a 0.2-μm polytetrafluorethylene (PTFE) syringe filter and then spin-coated onto the O_2_ plasma-treated substrate at 5000 rpm for 35 s. To form the In_2_O_3_ semiconductor layer, the spin-coated film was dried on a hotplate at 110 °C for 2 min and then annealed in a furnace at 350 °C for 2 h. For the bilayer case, the ZnO film was first formed on the O_2_ plasma-treated substrate. In detail, the 0.1 M ZnO precursor solution was filtered through a 0.2-μm PTFE filter and then spin-coated onto the O_2_ plasma-treated substrates at 4000 rpm. The spin-coated film was dried on a hotplate at 110 °C for 2 min and then annealed at the same temperatures as the In_2_O_3_. Subsequently, In_2_O_3_ was formed on the ZnO thin film by applying the same process as that used to produce the single-layer In_2_O_3_ film. To complete the TFT structure, 40-nm-thick Al source and drain electrodes were thermally deposited onto the semiconductor layer through a shadow mask; the channel width (*W*) and length (*L*) were 800 and 50 μm, respectively. Figure 2 shows schematic representations of the bottom-gate/top-contact TFT structures having the In_2_O_3_ and ZnO/In_2_O_3_ semiconductor layers.

The chemical characteristics of the oxide films were investigated using an X-ray photoelectron spectroscope (K-Alpha, Thermo Scientific, Waltham, MA, USA), and the crystallographic properties were characterized using an X-ray diffractometer (DMAX-2500, Rigaku, Tokyo, Japan). The surface morphologies of the films were examined using a field-emission scanning electron microscope (S-4300, Hitachi, Ibaraki, Japan). The electrical characteristics of the TFTs were evaluated using a semiconductor analyzer (4200-SCS, Keithley, Seoul, Korea).

## 3. Results and Discussion

**Figure 3:**
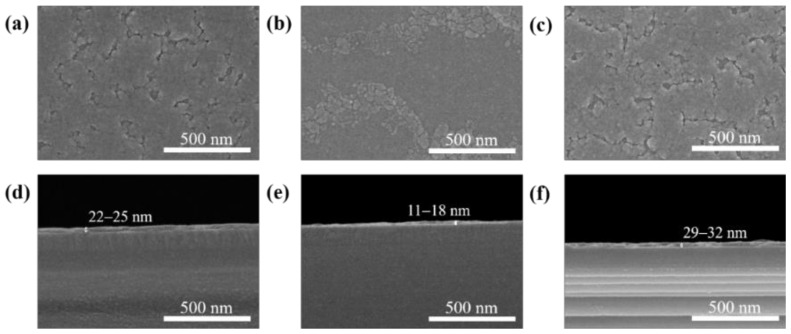
Surface and cross-sectional field-emission scanning electron microscopy (FE-SEM) images of the single-layer (**a**,**d**) In_2_O_3_ and (**b**,**e**) ZnO films, and (**c**,**f**) bilayer ZnO/In_2_O_3_ film.

Figure 3a–f shows the surface and cross-sectional FE-SEM images of the solution-processed In_2_O_3_, ZnO, and ZnO/In_2_O_3_ films, respectively. As shown in Figure 3a, the surface of the single-layer In_2_O_3_ film exhibits structural defects, such as cracks, voids, and grain boundaries. Figure 3b shows that the distribution of particles on the surface of the ZnO thin film is non-uniform and partially agglomerated. However, such particles on the surface of the underlying ZnO film barely affected the formation of the top layer In_2_O_3_ in the bilayer ZnO/In_2_O_3_ film. In fact, the surface morphology of the bilayer ZnO/In_2_O_3_ film shown in Figure 3c is similar to that of the single-layer In_2_O_3_ film shown in Figure 3a. Noteworthy are the grain boundaries in these films. The results suggest that grain boundary effects should be taken into account for analyzing the electrical characteristics of In_2_O_3_-based TFTs. From the cross-sectional FE-SEM images in Figure 3d–f, the thicknesses of the In_2_O_3_, ZnO, and ZnO/In_2_O_3_ films were approximately 22–25 nm, 11–18 nm, and 29–32 nm, respectively.

**Figure 4:**
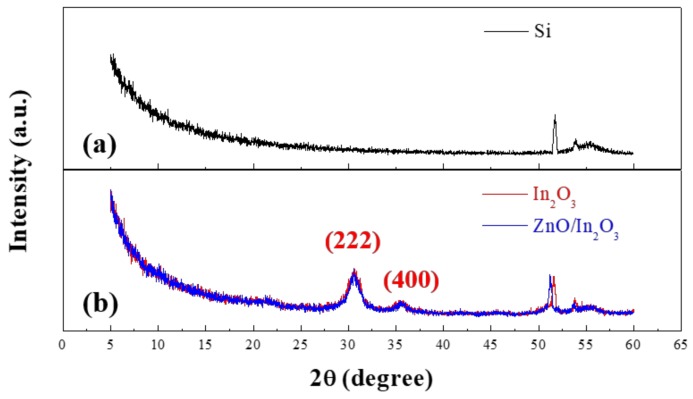
Grazing incidence X-ray diffraction patterns (GIXRD) patterns of (**a**) Si wafer; (**b**) single-layer In_2_O_3_, and bilayer ZnO/In_2_O_3_ films.

To investigate the crystallinity of the single-layer In_2_O_3_ and bilayer ZnO/In_2_O_3_ films, the prepared precursor solutions were spin-coated onto the Si substrates. Figure 4 shows the GIXRD patterns of the Si substrate and the prepared films. By varying the angles of incidence, we found four relatively sharp peaks on the prepared films. This indicates that the single-layer In_2_O_3_ and bilayer ZnO/In_2_O_3_ films exhibit a polycrystalline structure. Among them, the diffraction peaks appearing at 50–60° in Figure 4a coincide with the Si (100) substrate [19], while the two diffraction peaks appearing at about 30.6 and 35.5° in Figure 4b are the (222) and (400) peaks of In_2_O_3_, respectively [20]. The fact that diffraction peaks corresponding to In_2_O_3_ in the single- and bilayer films do not change significantly indicates that the crystallinity of the top layer In_2_O_3_ is not affected by the underlying ZnO film. However, no diffraction peak of crystalline ZnO is observed in the bilayer film, indicating that the solution-processed ZnO film had an amorphous structure in this study. 

**Figure 5:**
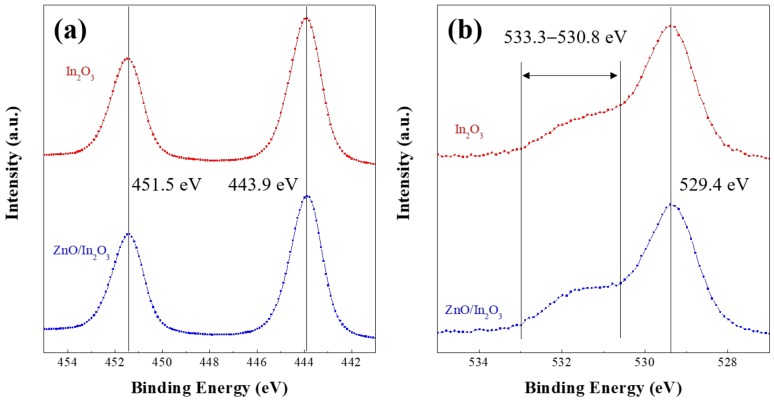
High-resolution X-ray photoemission spectroscopy (XPS) spectra of (**a**) In 3*d* and (**b**) O 1*s* orbitals in the single-layer In_2_O_3_ and bilayer In_2_O_3_/ZnO.

To understand the chemical characteristics of the prepared single- and bilayer films, the surface chemistry and electronic structure of the films were analyzed by using XPS. Figure 5a,b shows the high-resolution XPS spectra of the In 3*d* and O 1*s* orbitals in the single-layer In_2_O_3_ and bilayer ZnO/In_2_O_3_ films, respectively. As shown in Figure 5a, the two binding energy peaks of approximately 451.5 and 443.9 eV in the In 3*d* XPS spectrum are known to originate from the In 3*d*_3/2_ and In 3*d*_5/2_ orbitals, respectively [21]. The difference between these two binding energies corresponds to the spin-orbit splitting energy of 7.6 eV, suggesting that the valency of the indium in the film is mainly +3 [22]. We can see that the intensity and binding energy of the In 3*d* peak of the single- and bilayer do not change significantly. These results indicate that the chemical properties of the In_2_O_3_ film are not affected by the underlying ZnO film. Figure 5b shows the high-resolution O 1*s* XPS peaks of the single-layer In_2_O_3_ and bilayer ZnO/In_2_O_3_ films. The O 1*s* peak can be deconvoluted into two peaks in each curve. Among them, the lower binding energy peaks near 529.4 eV are attributed to the lattice oxygen [23,24]. It is known that the shoulders of the spectral region between 530.8 and 533.3 eV originate from the oxygen deficiency and the surface-absorbed oxygen species [23,24]. The comparable XPS results of both films indicate that the distribution of oxygen atoms or ions is not varied by the film structure. Accordingly, the XRD and XPS results confirm that there is no meaningful difference in the crystalline and chemical characteristics of In_2_O_3_ between the single-layer In_2_O_3_ and bilayer ZnO/In_2_O_3_ films.

**Figure 6:**
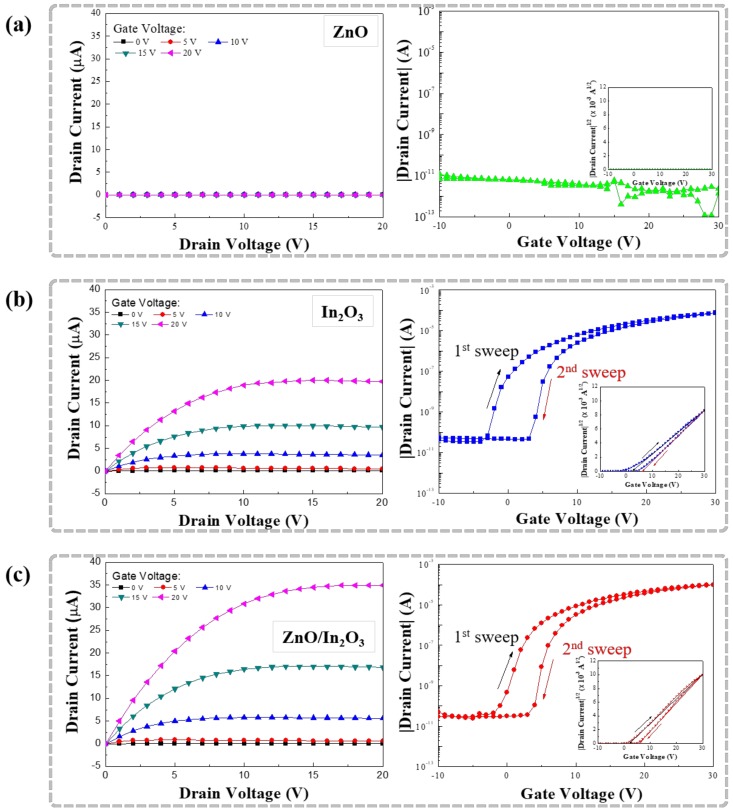
Electrical characteristics of TFTs fabricated with the (**a**) ZnO; (**b**) In_2_O_3_ and (**c**) ZnO/In_2_O_3_.

To investigate the difference in the level of performance of the TFTs, the output, transfer, and hysteresis characteristics of the transistors based on the single- and bilayer semiconductors were measured. Figure 6a–c shows the electrical characteristics of the ZnO, In_2_O_3_, and ZnO/In_2_O_3_ TFTs, respectively. Figure 6a shows that the single-layer ZnO TFT does not function as a TFT, primarily because of the very low thickness (approximately 11–18 nm) of the ZnO semiconductor layer prepared by the solution process. Similar results were found in other experiments upon the preparation of ZnO TFTs by the solution method [10]. On the other hand, the single-layer In_2_O_3_ and bilayer ZnO/In_2_O_3_ transistors exhibit typical operating characteristics of TFTs as shown in Figure 6b,c. The results indicate that the In_2_O_3_ serves as a semiconductor both in the single- and bilayer TFTs. In the measurement of the electrical characteristics of the TFTs, the output characteristics of the prepared TFTs were measured by changing the drain voltage (*V_D_*) from 0 to 20 V in increments of 1 V at different constant gate voltages (*V_G_*). The value of *V_G_* was increased from 0 to 20 V in increments of 5 V. It can be seen from the output characteristic curve that the In_2_O_3_ and ZnO/In_2_O_3_ TFTs possess excellent saturation characteristics. With the increase in *V_D_*, the drain current (*I_D_*) increases significantly, indicating that the devices possess significant *n*-type field-effect characteristics. Additionally, in the case of the bilayer ZnO/In_2_O_3_ TFT, the output currents improve under the same measurement conditions. The transfer characteristics were measured at a constant *V_D_* of 15 V, while the *V_G_* was reversibly swept from −10 to 30 V in increments of 1 V. The on/off current ratios (*I_on_*/*I_off_*), estimated from the *I_D_*–*V_G_* curves, were approximately 2.1 × 10^6^ and 3.4 × 10^6^ for the single-layer In_2_O_3_ and bilayer ZnO/In_2_O_3_ TFTs, respectively. Regarding the performance parameters obtained from the transfer characteristics, the field-effect mobility (*μ*) in the saturation region can be calculated by the following equation:

(1) $$I_{D}=\frac{W\mu C_{i}}{2L}{\left(V_{G}-V_{TH}\right)}^{2}$$

where *C_i_* is the capacitance per unit area of the gate dielectric layer and *V_TH_* is the threshold voltage. The *μ* values in the saturation region were approximately 0.3 and 0.5 cm^2^/Vs for the single- and bilayer TFTs, respectively. The *V_TH_* value was also extracted by linear extrapolation of the square root of the *I_D_*–*V_G_* curve. The *V_TH_* value (approximately 1.8 V) of the bilayer ZnO/In_2_O_3_ TFT was lower than that (approximately 2.4 V) of the single-layer In_2_O_3_ TFT; these values were taken from the transfer characteristics which were firstly measured by sweeping *V_G_* from −10 to 30 V. Accordingly, it is confirmed that the bilayer ZnO/In_2_O_3_ film contributes to enhancing the TFT performance in terms of drain current, field-effect mobility, and threshold voltage. However, the hysteresis phenomenon in the transfer characteristics of TFTs was not so reduced, even if the bilayer was used. From Figure 6b,c, the clockwise hysteresis is observed in the transfer curves both for the single-layer In_2_O_3_ and bilayer ZnO/In_2_O_3_ TFTs, which results in a *V_TH_* shift (Δ*V_TH_*) toward a positive direction upon reversing the *V_G_* sweep direction. The obtained Δ*V_TH_* values were approximately 3.9 and 3.8 V for the In_2_O_3_ and ZnO/In_2_O_3_ TFTs, respectively. Since grain boundaries in the solution-processed In_2_O_3_ semiconductor layer act as charge-trapping centers during TFT operation [25,26], the comparable Δ*V_TH_* values and clockwise hysteresis in our results are due to the polycrystalline nature of In_2_O_3_ observed in Figure 3 and Figure 4, not due to the influence of the bilayer structure. Table 1 summarizes the performance parameters of the fabricated TFTs.

**Figure 7:**
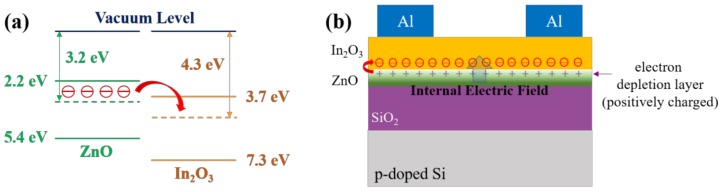
(**a**) Energy band structures of ZnO and In_2_O_3_ and (**b**) Description of internal electric field induced at the ZnO/In_2_O_3_ interface.

Since the crystalline and chemical characteristics of the top In_2_O_3_ layer in the bilayer structure are quite comparable to those of the single-layer In_2_O_3_ film, the enhancement in the electrical properties of the bilayer ZnO/In_2_O_3_ TFT may be explained with the band offset between ZnO and In_2_O_3_. Previously, it was reported that, when the vacuum level is set to 0 eV, the valence and conduction bands of In_2_O_3_ are positioned at deeper energy levels than those of ZnO [27]. This suggests that the band offset between ZnO and In_2_O_3_ may cause charge transport in the bilayer structure. Given that ZnO has a lower Fermi level (approximately 3.2 eV) than that (approximately 4.3 eV) of In_2_O_3_ [28,29], it is possible that electrons in the Fermi level of ZnO migrate to that of In_2_O_3_ in the bilayer structure, as depicted in Figure 7a. In consequence, the electron migration from ZnO into In_2_O_3_ will deplete the electrons near the surface of the underlying ZnO film and then the electron-depletion layer will be positively charged. Such an electron transport phenomenon in the bilayer structure thus forms an internal electric field at the ZnO/In_2_O_3_ interface, as shown in Figure 7b. A likely explanation is that the electron migration from the underlying ZnO film increases the carrier concentration in In_2_O_3_ and the internal electric field reduces the threshold voltage of the ZnO/In_2_O_3_ TFTs by being superimposed on the gate electric field. These behaviors effectively contribute to increasing the drain current and field-effect mobility. Consequently, the enhanced performance of the ZnO/In_2_O_3_ TFT can be understood through the electron transport between ZnO and In_2_O_3_ and the formation of an internal electric field in the bilayer ZnO/In_2_O_3_ structure.

**Figure 8:**
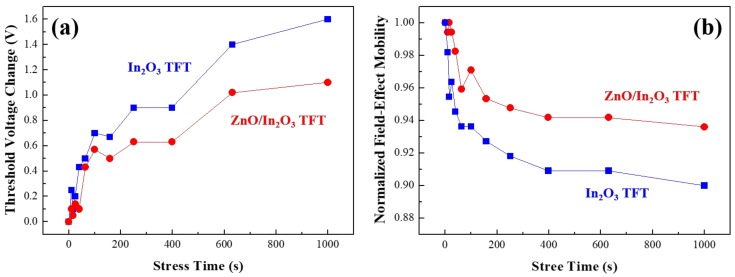
Variations in (**a**) threshold voltage and (**b**) field-effect mobility of the In_2_O_3_ and ZnO/In_2_O_3_ TFTs as a result of negative gate-bias stress.

In the literature, it was reported that the time for which TFTs in active-matrix displays are exposed to negative gate bias is more than 500 times longer than that under positive gate bias [30]. Taking into account the significance of negative bias stress, we subjected the single-layer In_2_O_3_ and bilayer ZnO/In_2_O_3_ TFTs to a negative gate-bias stress. For this experiment, a constant *V_G_* of −15 V was applied for an extended time period up to 1000 s, while the source was grounded, and the drain was set at 0 V. After applying a negative gate-bias stress for each stress time, the transfer characteristics of the In_2_O_3_ and ZnO/In_2_O_3_ TFTs were measured by changing *V_G_* from −10 to 30 V in increments of 1 V with *V_D_* fixed at 15 V. Figure 8a shows the changes in *V_TH_* according to the bias-stress time, which were obtained by subtracting the initial *V_TH_* (at stress time = 0 s) from the sequentially measured *V_TH_* values. The In_2_O_3_ and ZnO/In_2_O_3_ TFTs exhibited the positive values of the change in *V_TH_*, indicating that *V_TH_* was shifted toward a positive direction with proceeding the negative gate-bias stress. The positive shift of *V_TH_* can be ascribed to the influence of holes that are trapped in the TFT channel under a prolonged negative gate-bias stress [31,32]. Note that an additional positive voltage should be applied to the gate electrode to form an *n*-channel after repelling trapped holes, thereby causing a positive shift in *V_TH_*. Figure 8b shows the plots of normalized field-effect mobility *μ* versus bias-stress time; the obtained *μ* values were normalized with respect to the initial value. Clearly, the *μ* values of the In_2_O_3_ and ZnO/In_2_O_3_ TFTs decreased with a negative gate-bias stress. The decrease in *μ* elucidates that holes, which are trapped in the TFT channel during the negative gate-bias stress, deteriorate the electron transport. The most important observation here is that the positive shift in *V_TH_* and the decrease in *μ* were more pronounced in the single-layer In_2_O_3_ TFT than that for the bilayer case. Compared to the results of the single-layer In_2_O_3_ TFT, the small changes in *V_TH_* and *μ* of the bilayer ZnO/In_2_O_3_ TFT suggest that the internal electric field shown in Figure 7b is effective in reducing the negative gate-bias-induced field and suppressing the trapping of holes in the TFT channel. This explains how the internal electric field induced in the bilayer contributes to the electrical stability of transistors. However, the effects of air molecules on the electrical stability of the fabricated TFTs could not be explored in this study because the transistors were not passivated. To reach a more definite conclusion about the stability issues for the solution-processed bilayer ZnO/In_2_O_3_ TFTs, further studies should focus on the environmental stability of the transistors.

## 4. Conclusions

In summary, the single-layer In_2_O_3_ and bilayer ZnO/In_2_O_3_ TFTs were fabricated using the sol-gel spin-coating method, and the electrical characteristics of the resulting TFTs were investigated. From the GIXRD and XPS results, the intensity and position of the diffraction peaks related to In_2_O_3_ in the bilayer ZnO/In_2_O_3_ film were similar to those of the single-layer In_2_O_3_ film. This indicates that the underlying ZnO layer does not affect the crystallinity or chemical composition of the top In_2_O_3_ layer. In our results, the electrical characteristics of the TFTs were improved by using the bilayer ZnO/In_2_O_3_ semiconductor. Considering the energy bands of ZnO and In_2_O_3_, the enhancement in the performance of the bilayer TFTs, such as drain current, threshold voltage, and field-effect mobility, could be explained by the electron transport between ZnO and In_2_O_3_ and the formation of an internal electric field in the bilayer ZnO/In_2_O_3_ structure. In the negative gate-bias stress experiments, we found that the internal electric field helps to improve the electrical stability of the bilayer ZnO/In_2_O_3_ TFT by reducing the negative gate-bias-induced field and suppressing the trapping of holes in the TFT channel. However, the clockwise hysteresis and positive threshold voltage shift invariably occurred both in the single-layer In_2_O_3_ and bilayer ZnO/In_2_O_3_ TFTs owing to the grain boundaries in the In_2_O_3_ film. This means that grain boundaries in polycrystalline In_2_O_3_ thin films dictate the hysteresis behavior of these TFTs because they can act as trapping centers for mobile charge carriers. Consequently, this study demonstrates that the bilayer structure of solution-processed metal-oxide semiconductors is a viable means of enhancing the TFT performance. Since the rough surface of the underlying ZnO film may cause electron scattering in the TFT channel, we suggest that improving the surface morphology of the underlying ZnO films can further enhance the electrical properties of the solution-processed bilayer TFTs.

## Figures and Tables

**Table 1:** Performance parameters of the fabricated TFTs.

TFT	Threshold Voltage (V)	Mobility (cm^2^/Vs)	*I_on_*/*I_off_*	△*V_TH_* (V)
ZnO	-	-	-	-
In_2_O_3_	2.4 ± 0.1	0.3 ± 0.1	2.1 × 10^6^	3.9
ZnO/In_2_O_3_	1.8 ± 0.2	0.5 ± 0.1	3.4 × 10^6^	3.8
